# Salivary and serum alkaline phosphatase as biomarkers in type 2 diabetes mellitus: Association with periodontal health and disease

**DOI:** 10.6026/973206300214559

**Published:** 2025-12-15

**Authors:** Kinjal Desai, Bela Dave, Tanya Nagrani, Reena Chaudhary, Avani Patel, Shalini Gohil

**Affiliations:** 1 Department of Periodontology, Gujarat University, Ahmedabad, India; 2 Department of Periodontology, AMC Dental College and Hospital, Khokhara, Ahmedabad, Gujarat, India; 3 Department of periodontology & Implantology, Siddhpur Dental College and Hospital, Siddhpur, Gujarat, India; 4 Department of Oral & Maxillofacial Pathology, Siddhpur Dental College and Hospital, Siddhpur - 384151 Gujarat, India; 5 Department of Oral & Maxillofacial Pathology and Oral Microbiology, Narsinhbhai Patel Dental College and Hospital, Visnagar Sankalchand Patel University, Visnagar, Gujarat, India; 6 Department of Periodontology, Founder and chief dentist at Family Dental Clinic, Olpad, Surat, Surat - 395007, Gujarat, India

**Keywords:** Alkaline phosphatase, type-2 diabetes mellitus, chronic periodontitis, saliva, biomarker, periodontal disease

## Abstract

Chronic periodontitis in patients with type 2 diabetes mellitus (T2DM) requires reliable biomarkers for early detection and monitoring. Therefore, it is of interest to evaluate serum and salivary alkaline phosphatase (ALP) levels in 90 participants divided into healthy controls, diabetics with healthy periodontium and diabetics with chronic periodontitis. Salivary and serum ALP levels increased progressively across the groups and were significantly highest in diabetics with periodontitis (p < 0.001). Strong correlations were observed between salivary ALP, serum ALP and periodontal parameters such as probing depth and clinical attachment loss. Salivary ALP shows strong potential as a non-invasive biomarker for periodontal tissue destruction in diabetic patients.

## Background:

More than 460 million individuals throughout the globe suffer with Type-2 Diabetes Mellitus (T2DM), a long-term metabolic disease defined by high blood sugar levels caused by insulin resistance and relative insulin insufficiency [1]. Uncontrolled type 2 diabetes may have far-reaching consequences on many bodily systems, including the heart, kidneys, neurological system and eyes. The "sixth complication" of diabetes, periodontal disease, has only just been acknowledged in recent decades [2]. The periodontal ligament and alveolar bone, which support the teeth, are gradually eroded in periodontitis, an inflammatory disease that begins in dental plaque and is carried out by harmful bacteria [3]. Both type 2 diabetes and periodontitis are associated with an increased risk and severity of the disease; conversely, poor glycemic control due to severe periodontitis might worsen diabetic complications [4]. The underlying mechanisms involve a hyper inflammatory response, impaired wound healing and altered immune function in diabetic individuals, which amplify the tissue-destructive processes in the periodontium [5]. Because of this complex relationship, it is essential to diagnose diabetes early and monitor periodontal condition in patients in order to treat the illness effectively. Clinical parameters like as probing pocket depth (PPD) and clinical attachment loss (CAL) are traditionally used in periodontal diagnosis. However, these assessments only reveal disease activity that has already occurred, not inflammation that is occurring now or potential danger in the future. This has fueled the search for sensitive and specific biomarkers that can reflect the real-time pathological state of the periodontal tissues [6]. The use of saliva, often called a "mirror of the body's health" [7], as a diagnostic fluid has begun to show promise. Its non-invasive collection, ease of storage and rich composition of proteins, enzymes, hormones and genetic material make it an ideal medium for biomarker discovery and chair-side diagnostics [8]. Numerous salivary biomarkers have been investigated for periodontitis, including inflammatory cytokines, enzymes and microbial products. Among the potential enzymatic markers, alkaline phosphatase (ALP) has garnered significant interest. The liver, bile duct, kidney and bone all contain large amounts of ALP, an enzyme that hydrolyzes compounds by removing phosphate groups. Its serum concentration is a recognized indicator of biliary and bone diseases [9]. In the context of periodontitis, ALP is released from cells in the periodontium, including osteoblasts, fibroblasts and neutrophils, during active tissue inflammation and alveolar bone remodeling [10]. In otherwise healthy people, higher levels of ALP in saliva and gingival crevicular fluid (GCF) are associated with more severe periodontal damage [11, 16]. Therefore, it is of interest to compare the saliva and blood alkaline phosphatase levels of type 2 diabetic patients with and without chronic periodontitis to those of systemically and periodontally healthy persons, as well as to estimate these values.

## Materials and Methods:

After obtaining written informed agreement, 90 volunteers, ranging in age from 40 to 65, were included in the study.

They were allocated into three groups of 30 participants each:

[1] Group I (Healthy Controls): People without a history of type 2 diabetes who also healthy periodontium.

[2] Group II (T2DM with Periodontal Health): A healthy periodontium and a history of type 2 diabetes mellitus for at least a year constitute a patient population for this study.

[3] Group III (T2DM with Chronic Periodontitis): Diabetic patients with moderate to severe chronic periodontitis who have had a diagnosis of type 2 diabetes for at least a year.

## Inclusion and Exclusion Criteria:

People with type 2 diabetes who met the inclusion criteria for this study had a HbA1c level more than 6.5%, as set forth by the American Diabetes Association (ADA). Sites that were considered to have good periodontal health had the following characteristics: minimum gingival inflammation, no clinical attachment loss (CAL) and probing pocket depth (PPD) of 3 mm or less. The 2017 classification was used to identify chronic periodontitis, which included at least two non-adjacent locations with CAL measurements of >2mm, PPD 4mm or less and site with more loss of CAL was used to identify stages of periodontitis [15]. We excluded people who had type 1 or gestational diabetes, smoked or used tobacco products in the past, had a history of systemic conditions that affect ALP levels (such as liver disease, Paget's disease of bone, or renal failure), were pregnant or nursing, had taken antibiotics or anti-inflammatory drugs in the last three months, or had periodontal therapy in the last six months.

## Clinical examination:

The periodontal health of every individual was assessed by a single trained specialist. All teeth (with the exception of third molars) had the following clinical parameters recorded at six sites: mesio-buccal, mid-buccal, disto-buccal, mesio-lingual, mid-lingual and disto-lingual.

[1] Plaque Index (PI)

[2] Gingival Index (GI)

[3] Probing Pocket Depth (PPD) measured in millimeters (mm)

[4] Clinical Attachment Loss (CAL) measured in millimeters (mm)

All measurements were made using a UNC-15 periodontal probe (Hu-Friedy, USA). Other parameter like Radiographic parameter like OPG (ORTHOPANTOGRAM), Blood glucose estimation by glucometer was measured at the time of saliva collection and Physical parameters like age, gender, body mass index by weight & height of patients was also recorded.

## Sample collection:

[1] Collection of saliva for salivary alkaline phosphatase level estimation: The unstimulated whole saliva was collected using the 'spitting method'. All participants were requested to sit comfortably in an upright position and spit out the saliva accumulated in the mouth into an Eppendorf vial. 2-3ml of unstimulated saliva was collected. The saliva samples was first centrifuged at 3000 rotations per minute for 20 min to obtain a clear supernatant. The supernatant was used for ERBA Mannheim ALP reagent kit for the estimation of ALP levels with the help of micropipette in semi-automatic analyser.

[2] Estimation of the blood alkaline phosphatase and glycated Hemoglobin (HbA1c) Estimation: Under strict aseptic circumstances, 5 ml of venous blood was extracted from the antecubital fossa. Before centrifuging the blood at 3000 rpm for 15 minutes to extract the serum, it was left to clot for 30 minutes at room temperature. ERBA Mannheim Alkaline Phosphatase kit was used evaluate the blood alkaline phosphatase level in semi-automatic analyser. ERBA HBA1c test kit was used for HBA1C analysis in fully automatic analyser.

## Statistical analysis:

A software program developed by IBM Corp. in Armonk, NY, known as SPSS, version 25.0, was used for data compilation and analysis. The data was shown as the mean plus or minus the standard deviation (SD). We used the Shapiro-Wilk test to see whether the data was normally distributed. When comparing the three groups' means of clinical and a biochemical marker, a one-way analysis of variance (ANOVA) was used. For comparisons between pairs, Tukey's post hoc test was used if the ANOVA result was significant. The association between blood ALP, salivary ALP and other clinical indicators was determined using the Pearson correlation coefficient (r). It was deemed statistically significant if the p-value was less than 0.05.

## Results:

A total of 90 individuals, 48 of whom were male and 42 female, with an average age of 52.6 ± 6.8 years were involved in the research. All three groups had similar distributions of age and gender (p > 0.05), suggesting a representative sample. It was not surprising to see substantial group differences in clinical and glycemic markers (Table 1 - see PDF). Compared to Group I (5.2 ± 0.4%), the mean HbA1c values in Group II (8.1 ± 0.9%) and Group III (8.6 ± 1.1%) were substantially higher (p < 0.001). Group III's poor periodontal health was confirmed by their considerably higher mean PI, GI, PPD and CAL values compared to both Group I and Group II (p < 0.001). From Group I to Group III, there was a noteworthy and steady rise in the mean levels of ALP in both saliva and serum (Table 2 - see PDF). In Group III, the average salivary ALP level was 81.5 ± 12.2 U/L, which was noticeably more than in Group II (48.2 ± 9.5 U/L) and Group I (31.6 ± 7.9 U/L) (p < 0.001). Likewise, Group III had the highest mean serum ALP level (132.4 ± 18.5 U/L), as did Group II (95.8 ± 14.1 U/L) and Group I (72.4 ± 11.3 U/L) and these differences were statistically significant (p < 0.001). Comparing Group II to Group I, a significant, albeit lesser, increase in salivary and serum ALP was also seen (p < 0.05). For each of the 90 individuals, we ran a Pearson correlation analysis to see how salivary ALP related to the other characteristics (Table 3 - see PDF). Salivary ALP and serum ALP were shown to have a high and statistically significant positive connection (r = 0.78, p < 0.001). Additionally, the main periodontal markers, Mean PPD (r = 0.82, p < 0.001) and Mean CAL (r = 0.85, p < 0.001), showed a very substantial positive connection with salivary ALP. In addition, there was a somewhat favourable association (r = 0.59, p < 0.001) between the levels of salivary ALP and HbA1c.

## Discussion:

The complex interplay between T2DM and periodontitis highlights the urgent need for accessible and reliable diagnostic tools. As a biomarker indicating the periodontal state in T2DM patients, this research sought to evaluate the potential of salivary and serum ALP. Our findings demonstrate a significant, stepwise elevation in both salivary and serum ALP levels from healthy controls to T2DM patients with healthy gums and highest in T2DM patients suffering from chronic periodontitis. The most significant finding of our study was the markedly elevated ALP levels in Group III (T2DM with periodontitis). This is consistent with previous research that has independently linked high ALP levels to both periodontitis and T2DM. In periodontitis, the destruction of alveolar bone involves a dynamic process of resorption and formation. During inflammation, polymorphonuclear leukocytes (PMNs) and periodontal ligament fibroblasts produce ALP, an enzyme that is essential for bone production when secreted by osteoblasts [11, 12- 13]. Hence, it is probable that the elevated ALP levels in Group III are a result of both the severe inflammation of the gums and the dysregulated bone remodelling that is seen in periodontitis. It seems that this impact is amplified when type 2 diabetes is present. The periodontal host response is worsened by the pro-inflammatory milieu caused by the persistent low-grade systemic inflammation and oxidative stress intrinsic to type 2 diabetes [5]. This hyperinflammatory state can lead to greater cell turnover and tissue damage, resulting in a more substantial release of ALP into local fluids and the systemic circulation. This is supported by our observation that Group II (T2DM with healthy gums) had significantly higher ALP levels than the healthy controls (Group I). This suggests that T2DM itself, even in the absence of clinical periodontitis, can cause a subclinical inflammatory state that elevates basal ALP levels, a finding echoed by other studies investigating systemic markers in diabetes [14, 15].

A key aspect of our research was the direct comparison of salivary and serum ALP. The strong positive correlation (r = 0.78) found between the two is a critical finding. It suggests that salivary ALP levels reliably reflect systemic levels and, by extension, the underlying pathophysiology. This supports the "leakage" theory, where enzymes and other molecules from the serum can enter the oral cavity via the GCF and salivary glands and is also indicative of a local contribution from inflamed periodontal tissues [16]. The strong correlation validates the use of saliva as a non-invasive proxy for serum in monitoring ALP activity, which is a significant advantage in clinical settings, particularly for frequent monitoring of high-risk populations like diabetic patients. The correlation analysis further strengthened the diagnostic potential of salivary ALP. The very strong positive correlations with Mean PPD (r = 0.82) and Mean CAL (r = 0.85) indicate that as the severity of periodontal destruction increases, the level of salivary ALP rises proportionately. This dose-response relationship is a desirable characteristic for a biomarker, as it not only helps in differentiating health from disease but also in staging the severity of the condition. The moderate correlation with HbA1c (r = 0.59) reinforces the interconnectedness of glycemic control and periodontal inflammation, suggesting that elevated salivary ALP might reflect the dual burden of both uncontrolled diabetes and active periodontitis. Simple, non-invasive, chair-side salivary ALP test could potentially be developed to screen diabetic patients for underlying and often asymptomatic, periodontitis. Patients with elevated salivary ALP could be flagged for a comprehensive periodontal examination, facilitating early diagnosis and intervention. This would not only help in preserving oral health but could also contribute to better glycemic control by reducing the systemic inflammatory load originating from the periodontium. The research does have some drawbacks, however. We are only able to show a correlation since the cross-sectional design makes it impossible to prove causation. If ALP levels are a predictor of periodontitis start or progression in type 2 diabetic individuals, then longitudinal research is necessary to find out. The results may not be applicable to a broader population due to the limited sample size and the fact that they came from only one site, however they were sufficient for preliminary statistical analysis. These findings should be further validated by doing future studies with bigger, multi-center cohorts. Additionally, while we controlled for several confounders, other factors like diet and specific medications could potentially influence ALP levels.

## Conclusion:

Patients with type 2 diabetes and chronic periodontitis showed markedly elevated salivary and serum alkaline phosphatase levels, reflecting greater periodontal inflammation and tissue breakdown. The strong correlation between salivary ALP, serum ALP and clinical periodontal parameters highlights its diagnostic relevance. Salivary ALP emerges as a reliable, non-invasive biomarker with potential for routine screening and monitoring of periodontal disease in diabetic individuals.
